# Synthesis of halloysite nanotubes decorated with green silver nanoparticles to investigate cytotoxicity, lipid peroxidation and induction of apoptosis in acute leukemia cells

**DOI:** 10.1038/s41598-023-43978-y

**Published:** 2023-10-11

**Authors:** Xuan Zhang, Mostafa Heidari Majd

**Affiliations:** 1 Hematology Tumor Center, Xi’an People’s Hospital (Xi’an Fourth Hospital), Xi’an City, 710000 China; 2 Department of Medicinal Chemistry, Faculty of Pharmacy, Zabol University of Medical Sciences, Zabol, Iran

**Keywords:** Cancer, Cell biology, Chemical biology, Genetics, Molecular biology, Plant sciences

## Abstract

Leukemia is the 15th most common cancer in adults and the first most common cancer in children under the age of five, and unfortunately, it accounts for many deaths every year. Since leukemia chemotherapy usually fails due to chemotherapy resistance and disease relapse, many efforts are being made to develop new methods of leukemia treatment. Therefore, for the first time, we decorated halloysite nanotubes (HNTs) with green silver nanoparticles (Ag NPs) with the help of *Moringa Peregrina* leaves extract to increase the solubility of Ag NPs and to use the protective ability of HNTs against lipid peroxidation in erythrocytes. Cell survival assay by the MTT method showed that HNTs-Ag NPs can decrease the survival of Jurkat T-cells to about 10% compared to the control. The IC_50_ value was estimated as 0.00177 mg/mL after 96 h of treatment. Investigating the expression of genes involved in apoptosis by Real-time PCR proved that decorated HNTs with Ag NPs can increase the Bak1/Bclx ratio by 17.5 times the control group. Also, the expression of the caspase-3 gene has increased 10 times compared to the control. Finally, the reduction of malondialdehyde production after 24 h proved that the presence of HNTs can have a good protective effect on lipid peroxidation in erythrocytes. Therefore, on the one hand, we can hope for the ability of HNTs-Ag NPs to induce apoptosis in blood cancer cells and on the other hand for its protective effects on normal blood cells.

## Introduction

Halloysite nanotubes (HNTs) are inorganic natural aluminosilicates that can be used as biocompatible and sustainable excipients in the medical and chemical industries^1^. The hollow tubular structure of HNT with a lumen of 10–40 nm, an outer diameter of 50–70 nm, and a length of about 100–1000 nm has generated a growing research interest aimed at developing new organic or inorganic nanocomposites^2^. In the structure of this natural nanomaterial, the Al–OH groups are folded inside and the Si–O–Si groups are exposed to the outer surface so that at neutral pH, their inner surface has a positive charge and their outer surface has a negative charge^3,4^. Therefore, HNTs that possess large surface areas can capture drugs through adsorption to the outer walls or inside the tubules, or load drugs into the lumen or even between the layers^5^. So, depending on the molecular mass, the interactions between drugs and HNTs, and the solubility of drugs, drug release from HNTs may take several hours to fifteen days^6^. On the other hand, due to the high lumen volume, high length-to-diameter ratio, and better solubility, HNTs can be used as an ideal natural nano-carrier to increase the solubility of insoluble or poorly soluble compounds in water^3^.

Silver nanoparticles (Ag-NPs) may not have good solubility in water due to low surface charge or a size larger than 50 nm, and it is necessary to take measures for their solubility. In general, parameters affecting the dissolution of Ag-NPs include surface coating, temperature, size, morphology, ionic strength, and pH^7^. Also, by disrupting the surface charge responsible for electrostatic repulsion between NPs, the possibility of particle accumulation in biological fluids increases^8^. Peretyazhko et al. proved that changing the size and morphology of Ag-NPs can significantly affect their long-term stability in biological environments since small NPs release more Ag^+^ than large particles and are therefore more soluble^9^.

On the one hand, the positive effect of green synthesized Ag-NPs on cancer cells and the induction of apoptosis by them has been proven^10^, and on the other hand, the biocompatibility, low toxicity, safety and drug-carrying capacity of HNTs nanocarriers have been confirmed for the body^11^, since we decided, for the first time, to embellish and load of HNTs with Ag-NPs to increase the solubility and check their function on Jurkat floating cells. Because Jurkat cells are an important and widely used human T-lymphocyte cell line used to study acute T-cell leukemia and the expression of various receptors, we decided to use it for this study. This line is also used in research related to the effect of chemotherapy and radiation therapy on leukemia^12^. For this reason, Ag-NPs were biosynthesized directly on the surface of HNTs by *Moringa Peregrina* leaves extract to increase their stability in biological fluids and inhibit the Jurkat cells. *Moringa peregrina (Forssk.) Fiori* (A member of the *Moringaceae* family)^13^, which is found in different regions of Asia and Africa, is rich in protein, polyphenol, flavonoid, isothiocyanate, glycoside, triterpenoid, and essential amino acids^14^, and therefore we expect that biomolecules of its extract may cause a positive change in the expression of genes involved in apoptosis and be effective in the treatment of leukemia cells. On the other hand, due to the possible toxicity of Ag-NPs^15^, we will also investigate the oxidative stress caused by decorated HNTs on red blood cells.

## Experimental

### Materials

HNTs, trichloroacetic acid (TCA), thiobarbituric acid (TBA), and heparin were obtained from Sigma-Aldrich (Taufkirchen, Germany). Jurkat cells were ordered from the Research Institute of Biotechnology (Mashhad, Iran). Easy™ cDNA Synthesis kit was purchased from Pars Tous Biotechnology (Mashhad, Iran). High Pure RNA Isolation Kit was obtained from Roche (Rotkreuz, Switzerland). All chemical solvents and silver nitrate (AgNO_3_) were purchased from Merck (Darmstadt, Germany). All necessary cell culture media were purchased from either Gibco (Carlsbad, CA), Betacell (Mashhad, Iran), or Caisson labs (North Logan, UT).

#### Collection and extraction from *Moringa peregrina* leaves

After obtaining a permit from the environmental department of Iranshahr city (southeast of Iran) in Sistan and Baluchistan province, *Moringa peregrina* leaves were gathered from the outskirts of this city in autumn (permit number: 82411). This work was done in full compliance with environmental laws so as not to harm the nature of the area. They dried by lyophilization to preserve the bioactive compounds of the leaves. The identification and storage of the leaves were done by Dr. Behzad Zolfaghari at the Herbarium of Isfahan Faculty of Pharmacy with the code 2025. Afterward, 20 g of clean and fine powder of *Moringa peregrina* leaves were poured into a closed Erlenmeyer flask holding 50 mL of 70% v/v ethanol solution and put on an orbital shaker at a speed of 200 rpm at 30 °C for 120 h. The final mixture was centrifuged (for 6 min at 950 rpm), and the precipitate cleared. Then, the supernatant was dried using a freeze dryer at − 59 °C for 34 h and dissolved again in sterile deionized water. The aqueous extract was preserved at − 20 °C for further usage.

#### Synthesis of HNTs-Ag NPs

HNTs (100 mg, 0.34 mmol) were dissolved in 25 mL of sterile deionized water and sonicated for 22 min at 100% power using a probe sonicator. Then the solution was placed on a magnetic stirrer (50 °C and 875 rpm) and 50 mg of AgNO_3_ was added to it. After one hour, 10 mL of aqueous extract was added dropwise to the reaction mixture until its color changed to dark brown, indicating the production of HNTs-Ag NPs. A centrifuge (6 min, 950 rpm) was used to collect the final products. The supernatant was titrated with hydrochloride acid (10 mmol) to obtain the loading efficiency of Ag-NPs on the surface of HNTs.

#### Characterization of HNTs-Ag NPs

HNTs and HNTs-Ag NPs were characterized using Field Emission Scanning Electron Microscope (FESEM), Energy Dispersive X-ray (EDX), Zetasizer, and Infrared Fourier Transform (FT-IR) methods. For FT-IR spectroscopy, the samples were mixed with dry KBr at a ratio of 1:100 and turned into a fine and homogeneous powder. Then, the samples were made as thin disks under high pressure and transferred to the FT-IR instrument (Shimadzu IR PRESTIGE 21 spectrophotometer, Tokyo, Japan) and measured the vibration and rotation of molecules under the influence of infrared radiation in the region of 4000–500 cm^−1^. To measure zeta potential, 1 mg of HNTs and HNTs-Ag NPs separately were diluted in 5 mL of PBS at pH = 7.4. Then the zeta potential was determined using a particle size analyzer (Malvern Zetasizer_nanoZS, England).

For FESEM imaging, the sample fixes using double-sided conductive adhesive carbon tabs. In the next step, the gold coating was accomplished to make a conducting surface. After preparation, the sample was placed into the TESCAN MIRA3 XMU FESEM and photographed at 75 k× magnification. EDX analysis is an addition to FESEM to comprehend the chemical composition of HNTs and HNTs-Ag NPs, their elemental analysis as well as their distribution and concentration using X-ray energy.

#### MTT assay protocol to measure cell viability

The MTT method was used to measure cell viability and proliferation of Jurkat T-cells treated with HNTs-Ag NPs. For this purpose, Jurkat cells were propagated according to standard protocols for four weeks in cell culture flasks containing RPMI-1640 growth medium with 10% fetal bovine serum and 1% penicillin G-streptomycin until reached the desired number for MTT assay^16^. The cells at a density of 2 × 10^5^ cells/ml were incubated with six different concentrations of HNTs-Ag NPs (0.02 mg/ml to 2 mg/ml) in the growth medium for 48, 72, and 96 h. Each concentration was repeated three independent times (n = 3, ± SD) to reduce error. The supernatant was then carefully removed and replaced with 150 µL RPMI-1640 and 50 µL MTT in PBS and incubated for another 4 h. After that, the supernatant was carefully removed and the formazan crystals were dissolved by DMSO and prepared for spectroscopic analysis at 570 nm with ELISA Reader.

#### Examining genes involved in apoptosis induction and inhibition by real-time PCR

For this purpose, two flasks containing Jurkat T-cells with a seeding density of 5.0 × 10^6^ were prepared under standard conditions. 0.225 mg/mL (obtained from IC_50_) of HNTs-Ag NPs was added to one of the flasks, and no drug was added to the last one to be used as a control. After the 12 h treatment, cells were washed (2 times with PBS), and centrifuged (4000 rpm × 5 min) to collect cells. The supernatants were removed well and the cells were re-suspended in 200 µL PBS. Other steps of RNA isolation were performed according to the manufacturer’s protocol of the High Pure RNA Isolation Kit, which were fully described in our previous works^17–19^. Also, the details of cDNA synthesis using the cDNA synthesis kit were mentioned in our previous work^20^. The expression of the genes listed in Table 1 was measured using the Real-time PCR method and according to the appropriate thermal cycle by the Applied Biosystems StepOne™ device (Thermo Fisher Scientific, Massachusetts, USA)^17^.

**Table 1:** Primers used in real-time PCR.

Gene	Primer sequence
Bak1	Forward: TCTGGGACCTCCTTAGCCCT Reverse: AATGGGCTCTCACAAGGGTATT
Caspase-3	Forward: TGCAGTCATTATGAGAGGCAAT Reverse: AAGGTTTGAGCCTTTGACCA
Bclx	Forward: TGGAAAGCGTAGACAAGGAGA Reverse: TGCTGCATTGTTCCCATAGA
GAPDH	Forward: TTGCCATCAATGACCCCTTCA Reverse: CGCCCCACTTGATTTTGGA

#### Lipid peroxidation assay in erythrocytes

First, from the Zabol slaughterhouse, 500 ml of fresh goat blood was collected in heparinized tubes to prevent clotting (Code of ethics: IR.ZBMU.REC.1401.132). To obtain pure erythrocytes, 200 mL of blood was centrifuged twice for 30 min at 3000 rpm and the supernatant was discarded, then the erythrocytes were washed twice with PBS. A mixture containing 80 mL of PBS and 10 mL of erythrocytes was made and thoroughly homogenized by vortexing. Then, within this mixture, four different concentrations (1, 0.5, 0.25, 0.125, and 0 mg/mL) were prepared by the HNTs-Ag NPs with a final volume of 5 mL. At times 0 and 24 h, 1 mL of sample was removed and mixed with 1 mL of 20% TCA and 0.5 mL of TBA 0.028 M and incubated for 15 min at 100 °C, then centrifuged and the supernatant was separated. Finally, the absorbance of the supernatant solution was measured at a wavelength of 532 nm (the experiment was performed in triplicate (n = 3)).

## Results and discussion

### Characterization of modified HNTs

**Figure 1:**
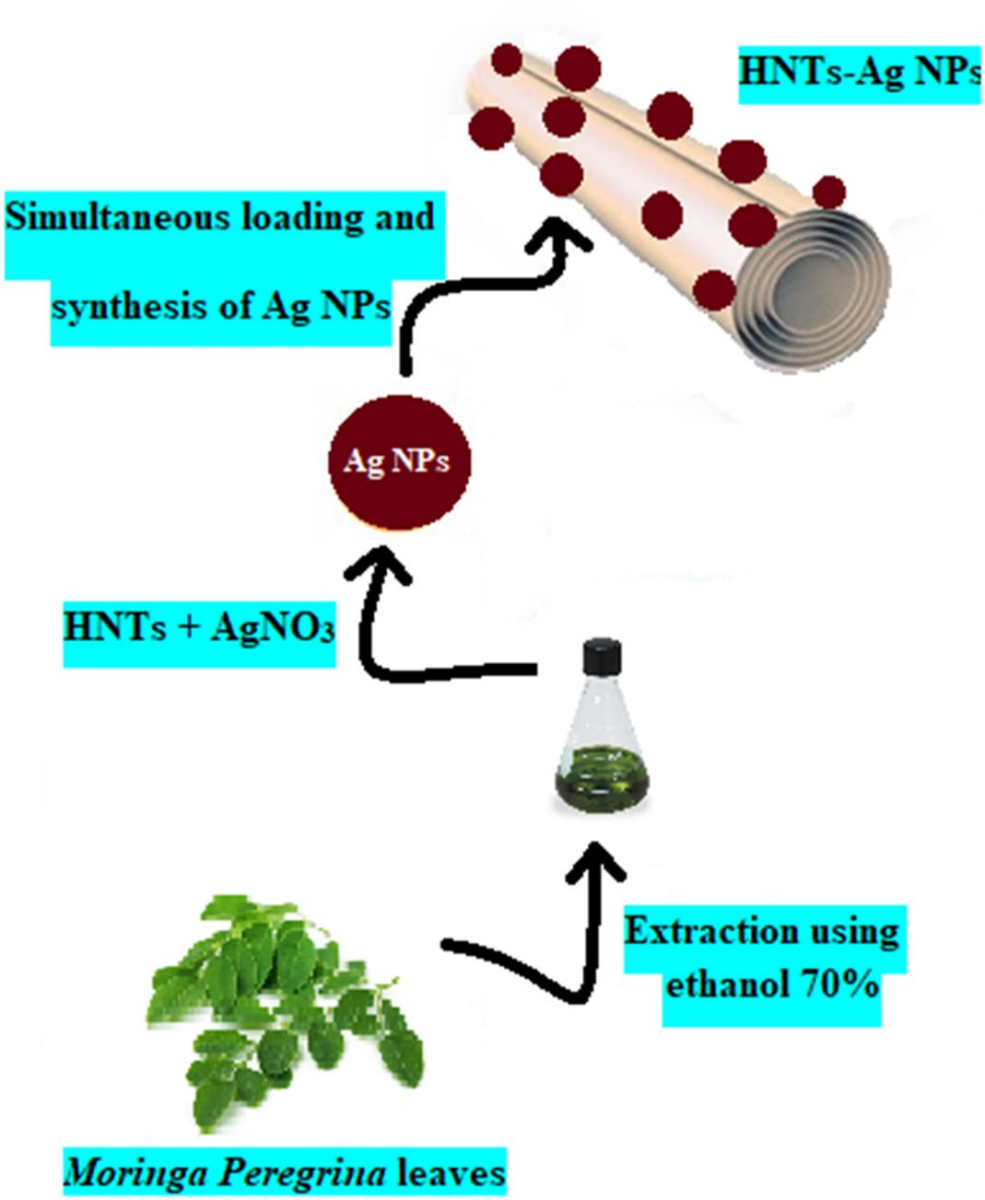
Schematic illustration the synthesis of HNTs decorated with green Ag NPs.

Halloysite nanotubes (HNTs) have attracted considerable attention in drug delivery due to their biocompatibility, physicochemical stability, and unique structural properties^4^. There are different methods for loading compounds inside the channel or on the surface (internal and external) of HNTs which can be mentioned as tubular encapsulation, surface adsorption, and surface modification^21^. In this research, using HNTs as a carrier, a type of silver-HNTs drug delivery system (HNTs-Ag NPs) was synthesized to deliver Ag-NPs to Jurkat cancer cells to increase the solubility and bioavailability of silver nanoparticles (Fig. 1).

**Figure 2:**
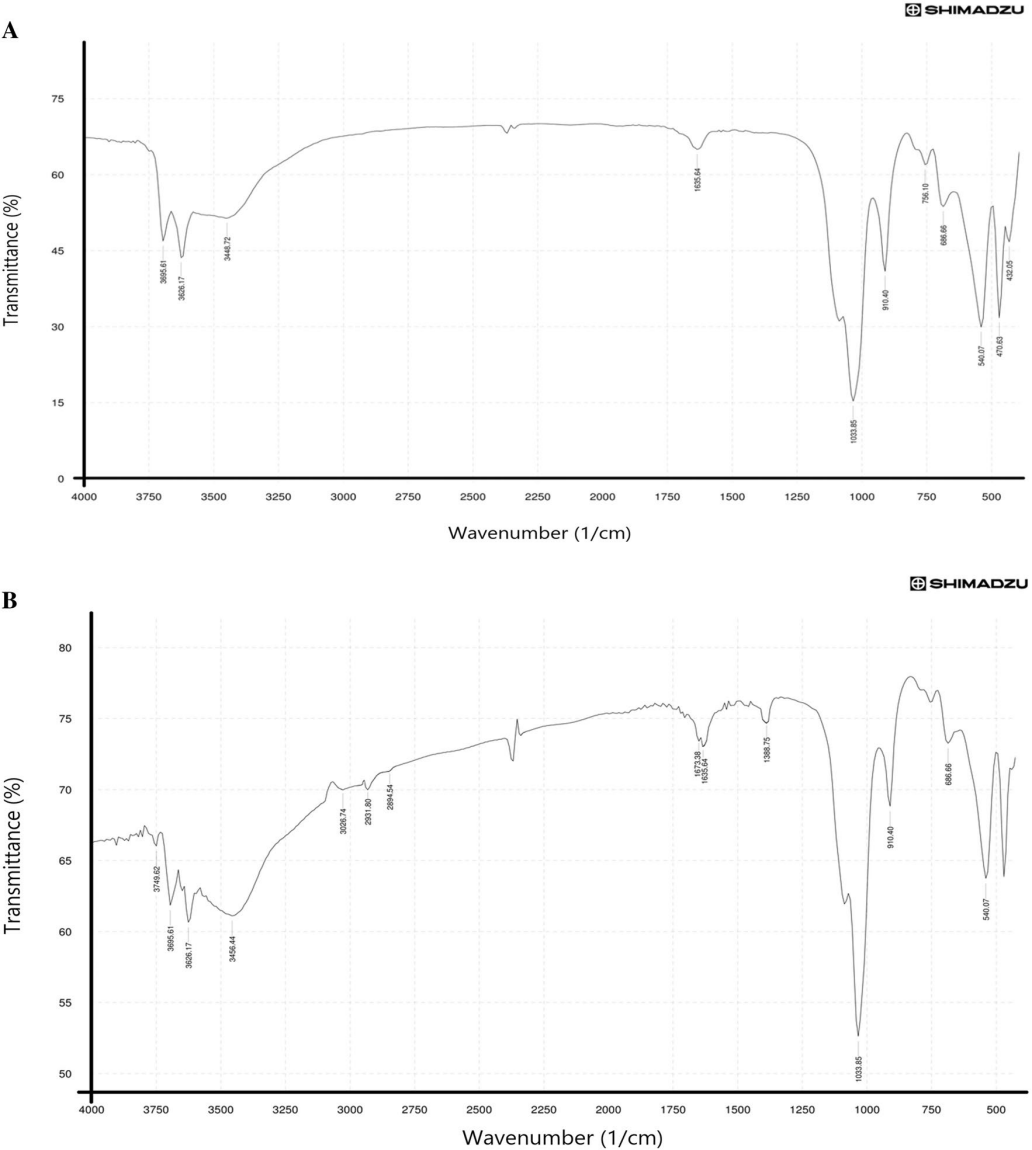
FT-IR spectra of (**A**) HNTs NPs and (**B**) HNTs-Ag NPs were taken using KBr with a resolution of 16 cm^−1^.

Accordingly, we decorated HNTs with green synthesized silver nanoparticles as an anticancer agent using *Moringa peregrina* leaves extract. Due to the negative charge of the outer surface of HNTs, the Ag^+^ ions in the silver nitrate solution can be absorbed on the surface using electrostatic attraction. By adding *Moringa peregrina* leaves extract, the initial color of the solution quickly changed from white (in the solution containing Ag^+^ ions and HNTs) to dark brown, which indicates the formation of Ag-NPs on the surface of HNTs due to the presence of reducing agents in the leaves extract. *Moringa peregrina* leaves are rich in polyphenols, protein, flavonoid, isothiocyanate, glycoside, triterpenoid, and essential amino acids^14^, which play a vital role in the reduction of Ag^+^ ions. To find out the correctness of the synthesis, the FT-IR spectrum of the obtained product (HNTs-Ag NPs) was taken and compared with the spectrum of HNTs. Common absorption bands between the two spectra of HNTs (Fig. 2A) and HNTs-Ag NPs (Fig. 2B) include the following, which can be seen in both. The absorption bands at 3695, 3626, and 910 cm^−1^, are respectively attributed to the stretching vibrations and the bending vibrations of the hydroxyl groups of the inner surface. The bands at 1033 and 470 cm^−1^ are due to the Si–O stretching and bending vibration, respectively. Also, the band at 540 cm^−1^ is related to the Al–O bending mode. Since the region of Ag NPs fingerprints is below 750 cm^−1^ and other metal oxides present in the HNTs-Ag NPs composition also vibrate in this region, it becomes difficult to detect it. Of course, the broadened and strengthened band at 3456 cm^−1^ (Fig. 2B) is related to the hydroxyl groups of the active molecules of *Moringa peregrina* leaves extract, which were involved in the reduction of Ag NPs. The absorption bands unique to HNTs-Ag NPs include the following (Fig. 2B). The asymmetric stretching vibrations bands (2894 and 2931 cm^−1^) related to the methylene groups of the active molecules have appeared in the spectrum and can prove the presence of Ag NPs on the HNTs surface. Also, the peak seen in the 3026 cm^−1^ is related to the unsaturated bonds of alkene (=C–H) that are present in the biomolecules in the leaves extract. In addition, the absorption bands at 1388 and 1673 cm^−1^ are due to C–N and C–O stretching, respectively.

**Figure 3:**
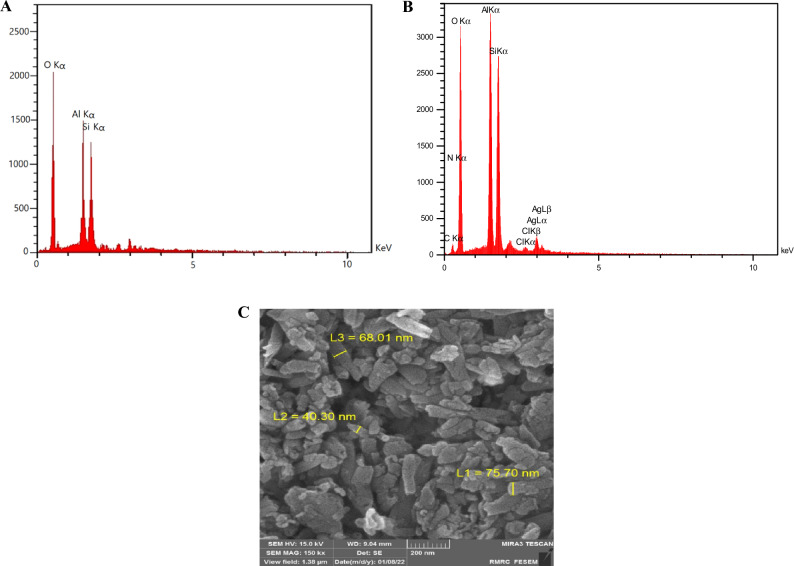
Energy-dispersive X-ray (EDX) spectroscopy shows the constituent elements of (**A**) HNTs and (**B**) HNTs-Ag NPs. (**C**) Using FESEM, the tubular structure of HNTs-Ag NPs with a diameter of about 61 nm and a length of about 160 nm is clearly visible.

Another method that can confirm the production of HNTs-Ag NPs is an elemental analysis by Energy-dispersive X-ray (EDX) spectroscopy. In previous studies, it was proved that HNTs are composed of three atoms of oxygen (O), aluminum (Al), and silicon (Si)^22^, after our investigation, it was determined by EDX analysis that its building blocks include 51.87% O, 24.37% Al, and 23.76% Si which is clear in Fig. 3A. But after decoration with green Ag NPs, additional peaks of carbon (C = 11.05%), nitrogen (N = 3.24%), and silver (Ag = 3.9%) confirm the presence of Ag NPs on the surface of HNTs-Ag NPs (Fig. 3B).

In Fig. 3C, the tubular morphology of HNTs-Ag NPs is clearly defined, so that their outer surface is mainly composed of silicon groups, and their inner surface is composed of aluminol groups. Also, the presence of Ag NPs on their surface is evident. With the help of the software available in the FESEM instrument, the average diameter of HNTs-Ag NPs was calculated to be about 61 nm. Of course, with the help of the scale embedded in the image, their average length was estimated to be about 160 nm.

**Figure 4:**
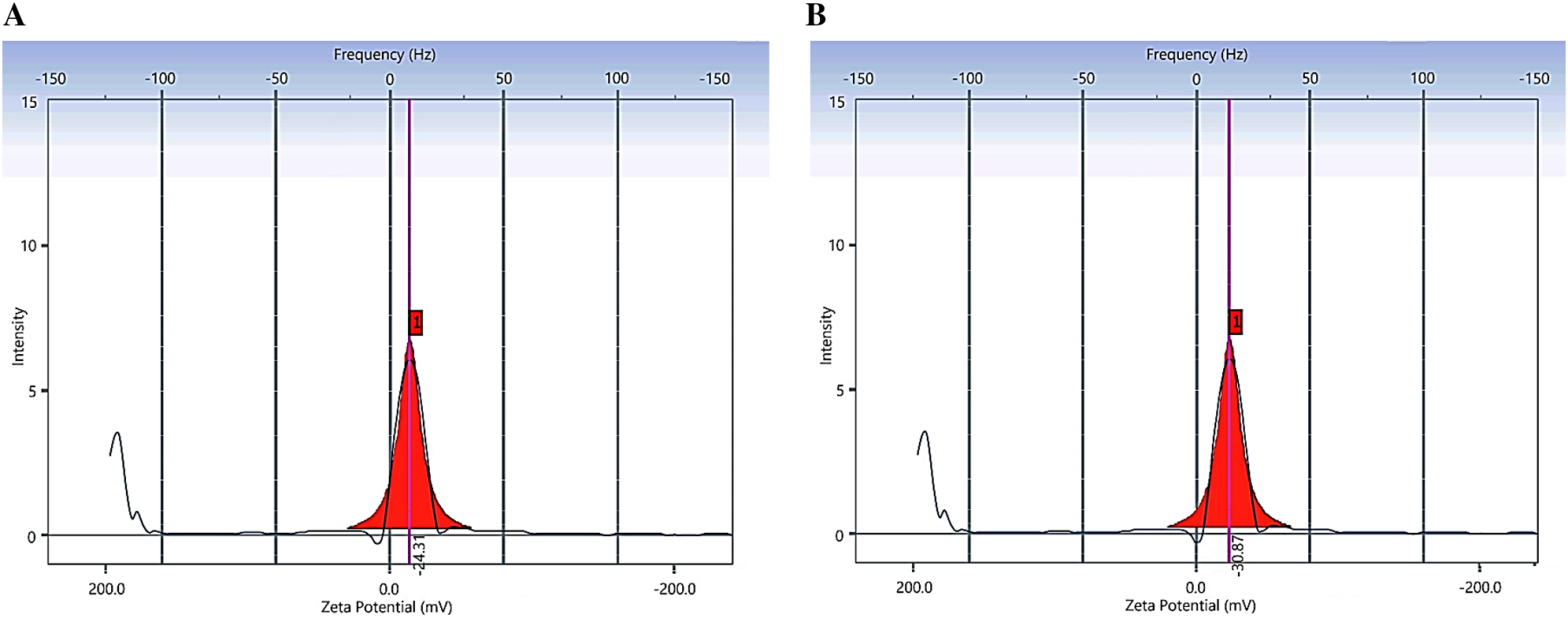
Surface potential of HNTs-Ag NPs was measured by Malvern Zetasizer_nanoZS.

It has been proven that the surface potential of HNTs at pH 7 is about − 22 mV^23^, so when Ag^+^ ions were added to the solution, can adsorb on the surface of HNTs and after adding *Moringa peregrina* leaves extract, quickly turned into Ag NPs and remain on the surface. Accordingly, HNTs and HNTs-Ag NPs were dispersed in PBS (Because blood pH is close to 7.40) and the zeta potentials were obtained around − 24 and − 31 mV respectively (Fig. 4A and B), which can be proof that Ag NPs have been adsorbed on the outer surface of HNTs and increased the initial negative zeta potential. Therefore, it can be hypothesized that initially the positive particles of Ag^+^ were trapped on the external surface and then the formed Ag NPs can be placed around this primary particle. In other words, a positive particle may exist as a linker between HNTs and green Ag NPs. It can also be concluded that the increase in negative charge is due to the fact that Ag NPs were created by the biomolecules of plant extract and their inherent surface charge is negative^24^. Therefore, we expect HNTs-Ag NPs to maintain their stability in biological environments for a long time and easily uptake by Jurkat T-cells.

### Measuring cell proliferation using MTT assay

It has been proven that Ag NPs can enter cancer cells through passive accumulation, which is called enhanced permeability and retention (EPR)^25^. Since passive targeting depends on the unique architecture of the tumor tissue as well as the shape, size, and capping agents applied on the surface of the NPs, we decided to increase bioavailability by loading Ag NPs onto modified HNTs as a carrier agent. Considering that our therapeutic target is the floating Jurkat T-cells, therefore the bioavailability, solubility, and systemic distribution of HNTs-Ag NPs are of particular importance.

**Figure 5:**
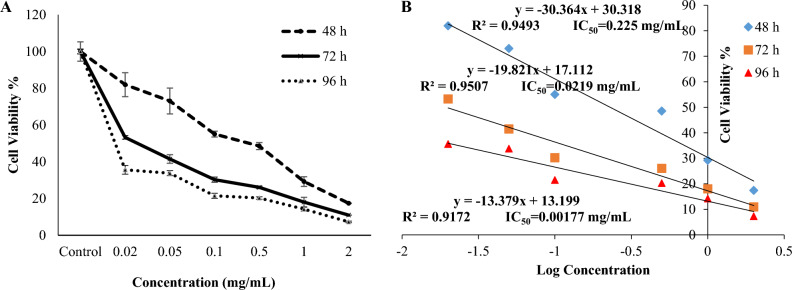
(**A**) Cell survival assay of HNTs-Ag NPs was performed using the MTT method. (**B**) The IC_50_ value was obtained by plotting a graph of cell viability (%) on the Y-axis and the concentration logarithm of the HNTs-Ag NPs on the X-axis, where Y was equal to 50 in the equation of the line.

As it is evident in Fig. 5A, the decoration of HNTs with Ag NPs followed by the increase in solubility was able to show a good effect on acute T-cell leukemia Jurkat cells. So, at the highest concentration and after 96 h of treatment with HNTs-Ag NPs, the cell viability decreased to about 7% of the control. The IC_50_ values in Fig. 5B confirm that HNTs-Ag NPs can effectively target leukemia cell lines in a concentration- and time-dependent manner.

### Expression level of genes involved in apoptosis induction and apoptosis inhibition

In 2020, leukemia is the 15th most common cancer and the 11th leading cause of cancer-related death worldwide^26^. More importantly, leukemia is the most common cancer in children under the age of five, and unfortunately, it accounts for many deaths every year^27^. Chemotherapy for leukemia usually fails, and unfortunately, a significant number of patients do not respond to it. Resistance to chemotherapy and recurrence of the disease are common obstacles in its treatment^28^. Therefore, many efforts are focused on preventing relapse, reducing drug side effects, and developing new methods for treating leukemia^29^. Many studies have shown that Ag NPs have promising anticancer effects and can induce cell apoptosis at low concentrations through mitochondrial-dependent and mitochondrial-independent pathways in various types of cancer cells^30^. Therefore, investigating the expression of genes involved in different pathways of apoptosis is used as a useful tool to reveal the molecular mechanisms of cytotoxicity.

Many studies have shown that Ag NPs can regulate the expression of Bax and Bcl-2 by producing reactive oxygen species (ROS). These two proteins play a key role in the regulation of apoptosis^31^ so the increase in Bax expression and the decrease in Bcl-2 expression indicate mitochondrial damage due to the treatment of cells with Ag NPs, which leads to the release of Cyt-c in the cytosol and subsequently the formation of apoptotic protease activating factor-1 (Apaf-1). Then, pro-caspase-9 is cleaved and produces the apoptosome, which ultimately leads to the activation of caspase-3 and -7 and induces apoptosis^32^. In a research conducted by EOM et al.^33^, it was found that Jurkat T-cells show great sensitivity to Ag NPs and they were inhibited at very low concentrations. However, the expression profile of genes such as p38, ERK, etc. showed that the expression of a small number of genes was changed by Ag NPs. Therefore, for the first time, we investigated the expression of genes involved in apoptosis in Jurkat T-cells that were treated with decorated HNTs by green Ag NPs.

**Figure 6:**
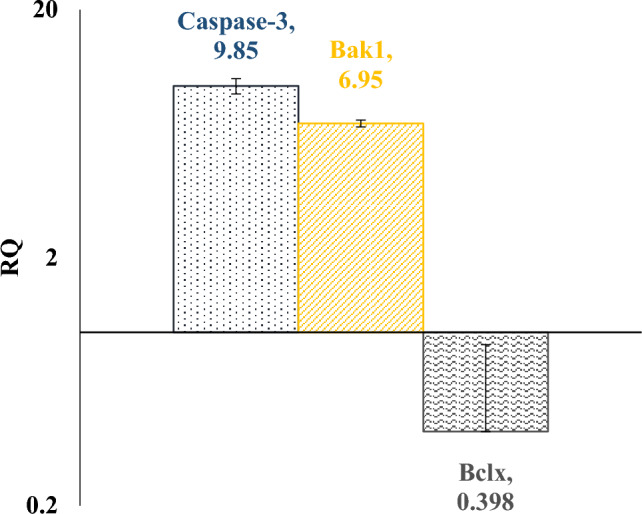
Show a logarithmic diagram related to gene expression involved in apoptosis. The baseline of the x-axis is equal to 1 and is related to the gene expression of the control group. Each sample was repeated four times, the average cycle threshold (CT) value was normalized to the housekeeping gene GAPDH, and the fold change expression was calculated by the ΔΔCT method. RQ is equal with ${2}^{-\Delta \Delta {\varvec{C}}{\varvec{T}}}$.

The results of Fig. 6 show that HNTs-Ag NPs were able to increase Bak1 gene expression about 7 times the control (the x-axis corresponds to the gene expression of the control group, which is equal to 1), while Bclx gene expression has decreased by about 0.4 of control. Therefore HNTs-Ag NPs increase the Bak1/Bclx ratio by 17.5 times compared to the control.

The extrinsic pathway of apoptosis was investigated by the level of caspase-3 gene expression. The results in Fig. 6 show that decorated HNTs can increase the expression of caspase-3 and thus can induce apoptosis through this pathway as well as the intrinsic pathway.

### Investigate the adverse effect of exposure to modified HNTs in erythrocytes

Approximately 10% of patients with acute leukemia develop the disease due to exposure to chemotherapy drugs, particularly alkylating agents or topoisomerase inhibitors, used to treat their primary cancer^34^. For this reason, NPs, which are used as drugs or drug carriers, should be evaluated in terms of possible risks to different body organs, especially blood ingredients. Because the unique structure and size of NPs during metabolism or as a result of exposure may lead to oxidative stress and cytotoxicity in erythrocytes^15^. A useful biomarker for oxidative stress is the production of malondialdehyde (MDA) by lipid peroxidation of unsaturated fatty acids, commonly measured by the thiobarbituric acid-reactive substance (TBARS) assay^35^.

On one hand, erythrocytes are prone to oxidative stress damage due to being exposed to a large amount of oxygen and having unsaturated fatty acids in the cell membrane, as well as not having an endoplasmic reticulum and nucleus to replace damaged proteins^36^. On the other hand, oxidative stress of various NPs, especially Ag NPs due to the presence of Ag^+^ ions, has been reported in various studies^37^. Various plant biomolecules such as terpenoids, saponins, polyphenols, antioxidant enzymes, and alkaloids are involved in the reduction reaction for the synthesis of green metal NPs. Also, glycoproteins, proteins, fatty acids, lipids, phenolics, sugars, and flavonoids strongly control the formation of free radicals and can easily play a role in eliminating oxidative stress^38^. Since *Moringa peregrina* leaves extract has large amounts of active biomolecules^39^, we expect that it will not harm erythrocytes.

**Figure 7:**
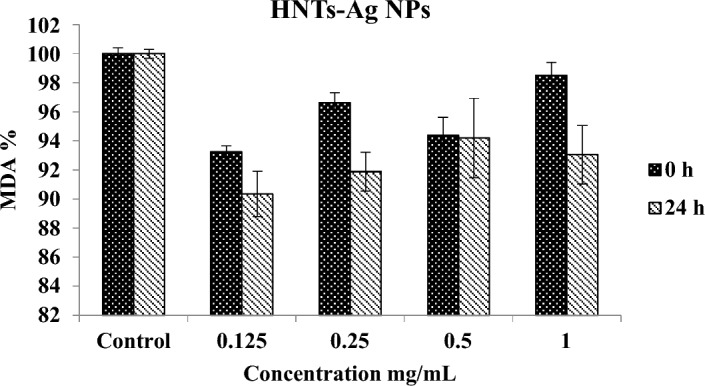
Investigation of MDA production by HNTs-Ag NPs after exposure to goat erythrocytes. For data analysis the one-way analysis of variance (ANOVA) was used, then results were estimated with Tukey's post hoc test (*P*-value < 0.05) n = 3, ± SD.

As shown in Fig. 7, HNTs-Ag NPs at the beginning of treatment (time 0) and at all concentrations, even at concentrations higher than IC_50_, do not increase MDA levels and do not cause oxidative stress on erythrocytes. More interestingly, after 24 h, all concentrations show protective effects and MDA levels decrease. This means that the long-term presence of decorated HNTs by Ag NPs not only does not cause lipid peroxidation but also has a protective effect and reduces oxidative stress. The reason for the decrease in the lipid peroxidation of decorated HNTs can be related to the anti-inflammatory properties of HNTs, where Cornejo-Garrido et al. proved that halloysite can reduce the production of nitric oxide and inhibit oxidative stress through lipid peroxidation^40^.

## Conclusion

Since leukemia treatment usually fails due to resistance to chemotherapy and disease relapse, we decorated HNTs with green Ag NPs with the help of *Moringa peregrina* leaves extract to increase their access to floating Jurkat T-cells. Cell survival assay showed that HNTs-Ag NPs can decrease Jurkat T-cell survival in a concentration- and time-dependent manner. Investigating the expression of genes involved in apoptosis by Real-time PCR proved that HNTs-Ag NPs can change the expression of genes involved in apoptosis both through intrinsic (the mitochondrial) and extrinsic apoptotic pathways. Finally, the reduction of MDA production after 24 h proved that the presence of HNTs can have a good protective effect on lipid peroxidation in erythrocytes. Therefore, on the one hand, we can hope for the ability of HNTs-Ag NPs to induce apoptosis in blood cancer cells and on the other hand for its protective effects on normal blood cells.

## Data Availability

The datasets generated during and/or analyzed during the current study are available from the corresponding author upon reasonable request.
